# Evaluation of a New Echocardiographic Tool for Cardiac Output Monitoring: An Experimental Study on A Controlled Hemorrhagic Shock Model in Anesthetized Piglets

**DOI:** 10.3390/jcm11185420

**Published:** 2022-09-15

**Authors:** Thibaut Markarian, Laura Grau-Mercier, Céline Occelli, Florian Ajavon, Pierre-Géraud Claret, Fabien Coisy, Xavier Bobbia

**Affiliations:** 1 Emergency Department, Timone University Hospital, Aix-Marseille University, 13005 Marseille, France; 2 Emergency Department, Nîmes University Hospital, Montpellier University, UR UM 103 IMAGINE, 30029 Nîmes, France; 3 Emergency Department, Pasteur 2 University Hospital, Nice Côte-d’Azur University, 06000 Nice, France; 4 Emergency Department, Montpellier University Hospital, Montpellier University, UR UM 103 IMAGINE, 34295 Montpellier, France

**Keywords:** shock, hemorrhagic, ultrasound, echocardiography, experimental, cardiac output

## Abstract

Background: Cardiac output (CO) monitoring is recommended in patients with shock. The search for a reliable, rapid, and noninvasive tool is necessary for clinical practice. A new echocardiographic CO flow index (COF) is the automatic calculation of the sub-aortic VTI multiplied by the automatic calculation of the heart rate (HR). The primary objective of this study was to show the correlation between COF and CO measured by thermodilution (COth) in a controlled hemorrhagic shock model in anesthetized piglets. Secondary objectives were to show the correlation between COth and CO calculated from left outflow tract (LVOT) measurement and manual VTI (COman), and CO measured by LVOT measurement and VTIauto (COauto). Methods: Prospective interventional experimental study. In seventeen ventilated and anesthetized piglets, a state of hemorrhagic shock was induced, maintained, then resuscitated and stabilized. The gold standard for CO and stroke volume measurement was thermodilution (COth). Results: 191 measurements were performed. The correlation coefficients (r) between COth and COF, COman, and COauto were 0.73 [0.62; 0.81], 0.66 [0.56; 0.74], and 0.73 [0.63; 0.81], respectively. Conclusions: In this study, the COF appears to have a strong correlation to the COth. This automatic index, which takes into account the HR and does not require the measurement of LVOT, could be a rapidly obtained index in clinical practice.

## 1. Introduction

Hemorrhagic shock results in a clinical state of acute circulatory failure, the main mechanism of which is a decrease in venous return due to a loss of circulating volume (internal or external loss of fluids) [1]. The goal of hemodynamic management in acute circulatory failure is to increase cardiac output (CO) to improve tissue perfusion. Optimal fluid management is an essential element of hemodynamic management in shock but remains a real challenge for clinicians. The main objective is to control hypovolemia and avoid hypervolemia. During shock management, CO and stroke volume (SV) measurements are recommended to evaluate the response to treatment, especially fluid therapy [2,3]. An increase of 15% of SV or CO is considered an indicator of good therapeutic response [4]. The gold standard for CO evaluation in critical care is transpulmonary dilution or thermodilution by central venous catheters (COth), which is an invasive technique [5]. Echocardiography is increasingly being used to replace invasive techniques in the hemodynamic assessment of critically ill patients, and it allows direct analysis of the main cardiac function parameters. Echocardiography is now acknowledged in emergency departments and critical care units for cardiocirculatory evaluation [6]. Sub-aortic velocity time index (VTI) is measured using pulse-Doppler in the outflow tract of the left ventricle with transthoracic echocardiography (TTE). This tool is reliable and has good intra- and inter-observer reproducibility [7]. VTI approximates the SV and, therefore, the CO [1]. The CO is estimated by the product of heart rate (HR), VTI, and the left outflow tract (LVOT) diameter measured in the parasternal long-axis view. Some studies have shown an important intra- and inter-observer variability of echocardiographic CO [8,9]. Those variations may be due to an imprecise LVOT diameter measurement. Indeed, inter-observer variability of the measurement site or probe orientation in LVOT measurement can lead to defective CO estimation. Thus, CO calculation by the conventional method is dependent on LVOT diameter calculation. This method exposes mistakes related to operator experience. This method also depends on the ultrasonographic acoustic window quality for VTI estimation. An automatized program for VTI measurement (VTIauto) provides a better correlation to invasive CO than manual (COman) measurement [10]. 

As the LVOT diameter is supposed to remain stable during hemodynamic variations of the same patient, a new echocardiographic index is proposed in this study: CO flux (COF), which is the automatized product of HR and VTIauto. This tool provides CO estimation without LVOT measurement, which could lead to lower inter-observer variability. Thus, COF differs from COman (calculated with manually measured LVOT diameter, VTI (VTIman), and HR) and COauto (calculated with manually measured LVOT diameter and HR, but automatically measured VTI (VTIauto)).

We hypothesized that COF is better correlated to COth than COman and COauto. 

The main aim of this study was to evaluate the correlation between COth and COF in a controlled hemorrhagic shock model in anesthetized piglets.

## 2. Methods

### 2.1. Materials

This study was designed as a prospective trial in a piglet model. The Animal Care and Use Committee of Languedoc-Roussillon (CEEALR-12013) approved the protocol, and all experiments were performed in an authorized animal research laboratory. This animal study was conducted according to the European Directive 2010/63/EC regulating the use of animals in science. All facilities and transport were compliant with current legal requirements. The experiments were carried out over four weeks at a rate of two experiments per day. The animals were kept in the laboratory for 1–4 days.

The primary aim was to evaluate the correlation between COF and COth in an experimental model of hemorrhagic shock (HS). 

Secondary outcomes were to study the correlation between:

− COth and COman: CO calculated with a manually measured sub-aortic velocity time index (VTIman) and LVOT;
− COth and COauto: CO calculated with VTIauto and manually measured LVOT;
− COF and stroke volume (SV) measured by thermodilution;
− VTIman and SV measured by thermodilution;
− VTIauto and SV measured by thermodilution;
− VTIman and COth;
− VTIauto and COth.

### 2.2. Animal Preparation

The animal preparation method we used has already been published [10,11,12]. Seventeen female piglets aged about three months were utilized. The piglets were premedicated by intramuscular injection of ketamine 10 mg.kg^−1^, atropine 0.05 mg.kg^−1^, and midazolam 1 mg.kg^−1^. Anesthesia was induced intravenously with propofol bolus (4 mg.kg^−1^) and cisatracurium (0.25 mg.kg^−1^). Anesthesia was maintained with propofol (8 mg.kg^−1^.h^−1^). After orotracheal intubation, the animals’ lungs were ventilated with an inspired fraction of oxygen of 0.21, a tidal volume of 8 mL.kg^−1^, and a positive end-expiratory pressure of 5 cm H_2_O. Once piglets were anesthetized, a 7-French double-lumen catheter was inserted, with ultrasound guidance, through the internal jugular vein into the right atrium. The central venous line was used to monitor central venous pressure and inject cold boluses for transpulmonary thermodilution. A 5-French arterial catheter with an integrated thermistor tip was inserted through the femoral artery (PiCCO^®^ Plus; Pulsion Medical Systems, Munich, Germany) and into the descending aorta for continuous arterial pressure monitoring, continuous pulse pressure variation (PPV) calculation, arterial blood sampling, and CO transpulmonary thermodilution measurement. The femoral vein was also cannulated with an 8.5-French catheter (Arrow^®^; Arrow International, Inc., Cleveland, OH, USA) for blood withdrawal and the administration of resuscitation fluids. All pressure-measuring catheters were connected to transducers (PiCCO^®^ Plus) for the continuous recording of systemic arterial pressure, HR, and temperature.

### 2.3. Experimental Protocol and Measurement Times

**Figure 1:**
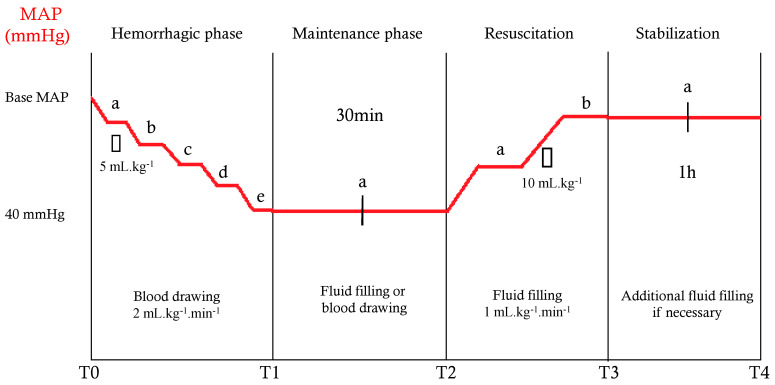
Study design. MAP = mean arterial pressure.

Our study design has already been published [10,11,12]. All steps are shown in Figure 1. T0 was the start of the experiment, and measured mean arterial pressure (MAP) was considered as the reference MAP. Several measurements were performed at T0: hemodynamic parameters (HR, MAP, systolic and diastolic arterial pressure, PPV, and COth), blood biochemistry (arterial lactate, arterial pH, and hemoglobin), and echocardiographic parameters (COF, VTIman, and VTIauto). Then, bleeding was performed by taking venous blood through the femoral venous catheter in increments of 2 mL.kg^−1^.min^−1^ until reaching an MAP of 40 mmHg, which defined T1. The same hemodynamic and echocardiographic parameters were measured at each 5 mL.kg^−1^ blood withdrawal (T0a, T0b, T0c, … to T1). In addition, biochemistry parameters were measured at T1. Then, the MAP was maintained between 35 and 45 mmHg during blood withdrawal or fluid filling for 30 min (T2). Hemodynamic and echocardiographic parameters were measured at 15 min (T2a), and all parameters were measured at T2. Piglets were then reanimated with an infusion of saline solution with a flow rate of 1 mL.kg^−1^.min^−1^. Hemodynamic and echocardiographic measurements were performed, each with 10 mL.kg^−1^ of fluid filling (T2a, T2b…), until the baseline MAP was reached (T3). Hemodynamic, echocardiographic, and blood chemistry measurements were collected at T3. The MAP was maintained at its baseline value ± 10% by fluid infusion for an additional hour (T4); then, all animals were sacrificed using i.v. thiopental infusion (2 g). Hemodynamic and echocardiographic parameters were measured at 30 min (T3a), and all parameters were measured at T4.

### 2.4. Echocardiography

Two emergency physicians with intensive care unit experience performed and evaluated the echocardiogram videos; both have obtained an academic degree in ultrasound applied to critical care. Echocardiography was performed with a Venue R2.6 ultrasound device (Venue R2.6, GE Medical Systems, Milwaukee, WI, USA) equipped with a phased array transducer (GE 3Sc-RS Phased Array Probe, GE Medical Systems, Milwaukee, WI, USA). Cardiac ultrasound was performed following guidelines from the American Society of Echocardiography [13]. The VTIman was computed using the average of three successive measures on an apical five-chamber view using pulse-Doppler in the LVOT. Using artificial intelligence, the ultrasound device optimized the pulsed-Doppler box place, recorded four seconds of the Doppler spectrum, traced the outline of VTIs, and averaged all VTIs of the recording to obtain the VTIauto [10]. HR was automatically averaged over the measurement time of the VTIauto, allowing automatic calculation of COF. The LVOT diameter was measured at T0, enabling calculation by the ultrasound device of COman and COauto. The success rate was calculated for each method (manual and automatic) based on its ability to obtain a measurement.

### 2.5. Statistical Analysis

Quantitative data were expressed as medians with 25th and 75th percentiles (25th percentile–75th percentile). Qualitative variables were expressed as frequencies with percentages. Correlation coefficients were calculated to assess the relationship between quantitative variables. The significance level was set at 5% for all tests. Statistical analysis was performed under R 4.0.2 (2020, R Foundation for Statistical Computing, Vienna, Austria).

## 3. Results

### 3.1. Animals and Compliance to the Protocol

Seventeen animals were included. A targeted MAP of 40 mmHg was successfully achieved and maintained for 30 min for all piglets. They were all females. The median weight was 30 kg [30; 33]. Table 1 shows the hemodynamic and biological characteristics of the piglets.

### 3.2. Measures

**Figure 2:**
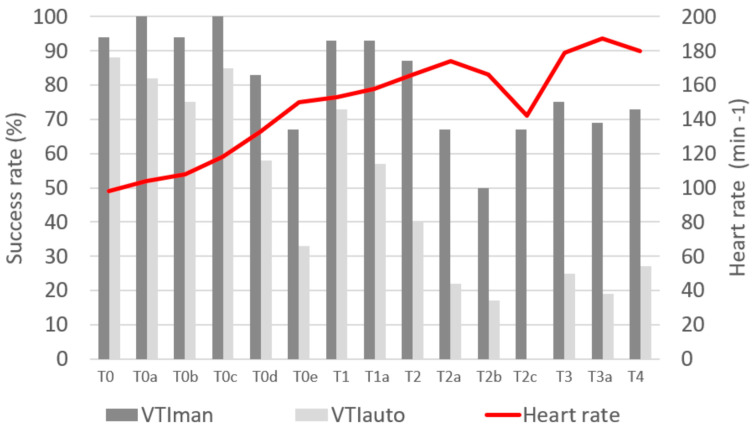
Feasibility according to the experimental time of manually measured sub-aortic velocity time index (VTIman) and automatically measured sub-aortic velocity time index (VTIauto).

**Figure 3:**
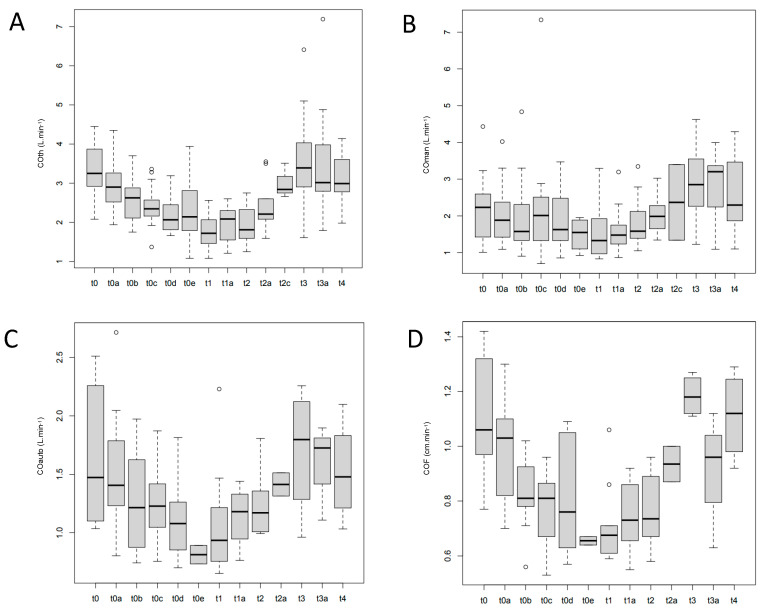
Variations of CO (L.min^−1^) during the experiment. (**A**) CO as measured by thermodilution (COth); (**B**) CO calculated by ultrasound device with a manually measured sub-aortic velocity time index (COman); (**C**) CO calculated by ultrasound device with an automatically measured sub-aortic velocity time index (COauto); (**D**) CO flux (COF).

In total, 191 measurements were recorded: 161 measurements of VTIman were successfully performed (84%), and 101 for VTIauto and COF (53%). In addition, LVOT measurements were successfully performed in all piglets. Figure 2 shows the feasibility at each experimental time. Variations of COth, COman, COauto, and COF during the experiment are shown in Figure 3.

### 3.3. Outcomes

**Figure 4:**
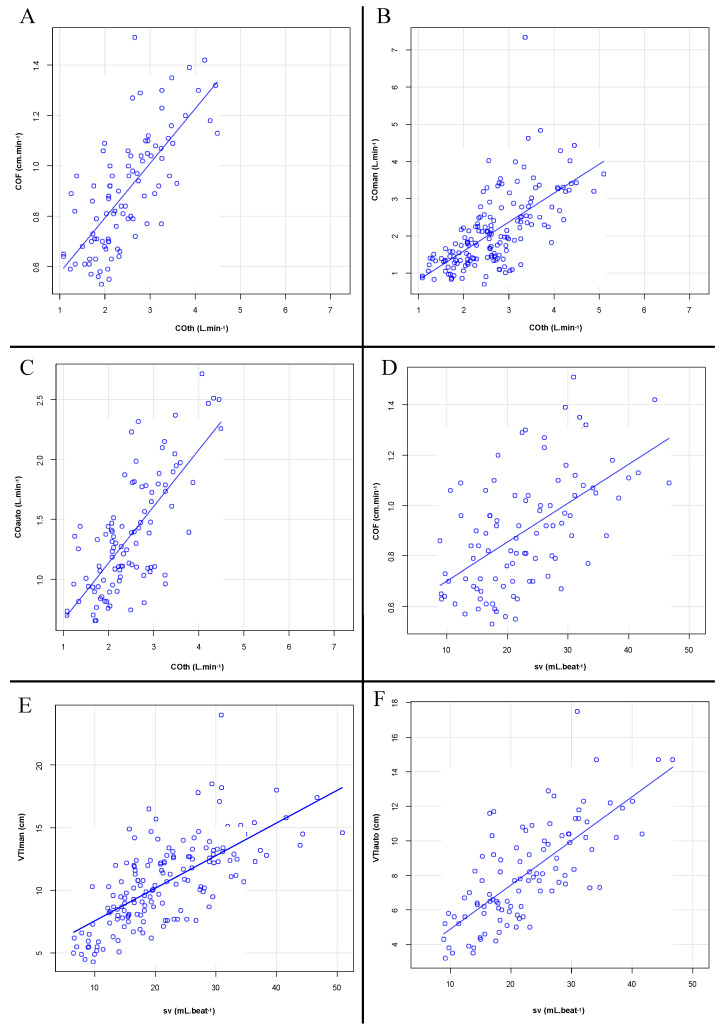
Scatter plots showing the correlations between CO measurements. (**A**) CO as measured by thermodilution (COth) and CO Flux (COF); (**B**) COth and CO calculated by ultrasound device with a manually measured sub-aortic velocity time index (COman); (**C**) COth and CO calculated by ultrasound device with an automatically measured sub-aortic velocity time index (COauto); (**D**) stroke volume (SV) and COF; (**E**) SV and a manually measured sub-aortic velocity time index (VTIman); (**F**) SV and an automatically measured sub-aortic velocity time index (VTIauto).

The correlation coefficients (r) between measures for the primary and secondary objectives are shown in Table 2. Figure 4 shows the correlation of straight lines.

## 4. Discussion

This study describes the correlation of a new automatic echocardiography index (COF) with COth in a model of controlled hemorrhagic shock. As a primary result, COF was strongly correlated to COth (r = 0.73). In a hemorrhagic model, COth is a reliable tool to follow blood volume variations, and COF decreases in response to blood removal and increases during blood volume compensation (Figure 3).

The search for noninvasive techniques to evaluate CO is ongoing in the literature [14,15,16,17,18], especially in anesthesiology and critical care [19]. Echocardiography is a noninvasive technique that is now widely used in emergency departments and critical care units for cardiocirculatory evaluation [6]. Feissel et al. found that peak aortic blood flow velocity is a valuable method for predicting the hemodynamic effects of volume expansion in septic shock patients receiving mechanical ventilation [20]. It is also established that VTI approximates the SV and, therefore, the CO [1]. However, the echocardiographic calculation of CO by VTI measurement is exposed to several biases: calculation error from the angle of insonation, difficulty in assessment of the VTI, problems with correct measurement of the LVOT diameter, incorrect HR measurement, or averaging enough VTI.

In 2019, our team demonstrated that an automatic program for VTI measurement (COauto) was better correlated (r = 0.83) with COth than manual (COman) measurement (r = 0.54) in a piglet model [10]. Our results are concordant with a strong correlation between COth and COauto (r = 0.73) and a moderate correlation with COman (r = 0.66). Several articles [8,9] have shown a lack of accuracy in manual measurements of VTI to estimate CO. 

A recent study of mechanically ventilated patients showed a correlation coefficient of 0.64 between VTI (calculated by averaging five VTI) and SV [8]. In the present study, the correlation of VTIman and VTIauto with SV was stronger, with values of 0.70 and 0.73, respectively. Another study performed in a cardiology setting showed a correlation of 0.50 between a cardiac index evaluated by VTI (calculated by averaging three VTIs) and one measured by thermodilution [14]. If the VTIauto system can correctly trace VTI contours, the good results of COauto may be due to the device’s capacity to average many VTIs [10]. The VTIauto is an average of all VTI recordings during a 4 s period. According to the HR in our model (Table 1), the averages of 6–10 VTIs were measured. Indeed, the HS model induced a high respiratory variation of VTI [10]. The averaging of many VTIs is probably the most important in hypovolemia conditions.

It seems established that COauto improves the estimate of COth with the automation of VTI measurement, but LVOT measurement remains manual. Inter- and intra-observer variability in LVOT diameter was estimated by Shiran et al. at about 4.8 ± 4.1 and 2.8 ± 1.9% for TTE, and 4.2 ± 3.1 and 2.5 ± 1.6% for transesophageal echography, respectively [21]. They also showed that the diastolic LVOT diameter by transesophageal echography was slightly smaller compared with the systolic LVOT diameter (−0.03 ± 0.07 cm) [21]. Assuming LVOT diameter remains stable during blood volume variations, excluding this measurement for cardiac output estimation seems feasible. In addition, COF, the automatized product of HR and VTIauto, allows for a more accurate estimation of HR because it is averaged throughout VTIauto recording and provides a significant time gain. In this model, COF had the same correlation with COth as COauto. The use of an experimental model could explain this result. The inter- and intra-observer variability of LVOT diameter in piglets are unknown; hence, we cannot compare measurement errors in human and pig models. COF is interesting because it seems to be as accurate as COauto, but with time saving linked to the absence of LVOT diameter measurement. The advantage of COF over COauto due to inter- and intra-observer LVOT measurement variability remains unproven. 

In the present study, VTIauto suffers from moderate feasibility, with a global success rate of 53%. However, the success rate drops under 20% in critical conditions (Figure 2). Consequently, the feasibility of COF is the same. In a previous study [10], VTIauto feasibility was higher, around 60%, which could be due to the operator’s performance or piglets’ inherent characteristics, as the same ultrasound device was used. Because the measurement of CO is important in highly critical conditions, this will have to be improved. In the present model, HR can increase above 200 beats min^–1^, and CO can decrease to less than 1.5 L.min^–1^ (Table 1). Because these values are less extreme in humans, it is possible that COF is more feasible in critical clinical conditions.

### Limitations

First, COF is compared to COth, which approximates CO with thermodilution. If COth is the reference technique in clinical conditions, then in experimental conditions, a flow meter around the pulmonary artery or aorta could provide a more precise measurement of CO. Second, even the COF discard the volumetric dimension (cardiac output measured by echocardiography is dependent on valve area measurement) and is not equal to cardiac output for a specific pig, because the aortic valve area is constant, the COF varies and track cardiac output trend during the hemorrhagic shock model. Third, TTE measurements were performed by two experienced physicians. Feasibility and variability of measurement could have been worse with non-expert physicians. Fourth, this model provides shock in a strictly hypovolemic pathway, whereas hemorrhagic shock may result from a combination of mechanisms. For example, decreased venous return due to a loss of circulating volume may be associated with a failure of the heart’s pump function (blunt cardiac injury) or a loss of vascular tone (spinal injury). Finally, measures were performed in a piglet model with standards of vital constants that differ from humans in critical conditions. For both reasons, clinical studies in critical conditions are necessary to assess the relevance of COF to estimate CO.

## 5. Conclusions

In this controlled hemorrhagic shock model, COF is strongly correlated with COth as well as COauto, but without the need to measure LVOT. Manual VTI and CO measurements are less correlated with thermodilution, confirming the need for using an automatic measurement of VTI when feasible. Taking into account the SV and HR without the need for LVOT measurement, COF seems to be a fast, reliable, and noninvasive tool for evaluating CO. Its clinical interest should be studied in critical conditions.

## Figures and Tables

**Table 1:** Hemodynamic and biological data at baseline (T0), hemorrhagic phase (T1), maintenance phase (T2), resuscitation phase (T3), and stabilization phase (T4).

Median arterial pressure (mmHg)	
T0	73 [69; 80]
T1	39 [36; 40]
T2	41 [39; 43]
T3	80 [68; 88]
T4	67 [62; 72]
Heart rate (min^–1^)	
T0	102 [80; 120]
T1	163 [138; 172]
T2	190 [166; 194]
T3	172 [169; 202]
T4	180 [162; 201]
COth (L.min^–1^)	
T0	3.15 [2.99; 3.59]
T1	1.63 [1.42; 2.02]
T2	1.87 [1.66; 2.30]
T3	3.42 [2.51; 4.89]
T4	2.80 [2.58; 3.90]
Hemoglobin (g.dL^–1^)	
T0	9.5 [8.8; 9.9]
T1	8.2 [7.5; 8.9]
T2	7.5 [6.9; 8.3]
T3	6.9 [4.3; 7.6]
T4	6.5 [5.5; 7.8]
pH	
T0	7.45 [7.39; 7.48]
T1	7.44 [7.39; 7.47]
T2	7.36 [7.07; 7.42]
T3	7.32 [7.27; 7.38]
T4	7.35 [7.34; 7.40]
T0–T1 volume of hemorrhage (mL.kg^–1^)	−25 [−33; −14]
T1–T2 volume for maintenance (mL.kg^–1^)	2 [−6; 5]
T2–T3 resuscitation fluid volume (mL.kg^–1^)	16 [13; 33]
T3–T4 volume for stabilization (mL.kg^–1^)	3 [0; 11]

Data are expressed as median [Q1; Q3]. COth: Cardiac output measured by thermodilution.

**Table 2:** Correlation coefficients between measures for the primary and secondary objectives.

Measure 1	Measure 2	Correlation Coefficient [95%CI] between Measures 1 and 2	*p*
COth	COF	0.73 [0.62; 0.81]	<0.01
	COman	0.66 [0.56; 0.74]	<0.01
	COauto	0.73 [0.63; 0.81]	<0.01
	VTIman	0.54 [0.42; 0.64]	<0.01
	VTIauto	0.45 [0.28; 0.59]	<0.01
SV	COF	0.57 [0.41; 0.69]	<0.01
	VTIman	0.70 [0.59; 0.75]	<0.01
	VTIauto	0.73 [0.63; 0.81]	<0.01

CO as measured by thermodilution (COth); CO Flux (COF); CO calculated by ultrasound device with a manually measured sub-aortic velocity time index (COman); CO calculated by ultrasound device with an automatically measured sub-aortic velocity time index (COauto); stroke volume (SV); manually measured sub-aortic velocity time index (VTIman); automatically measured sub-aortic velocity time index (VTIauto).

## References

[1] Cecconi M., De Backer D., Antonelli M., Beale R., Bakker J., Hofer C., Jaeschke R., Mebazaa A., Pinsky M.R., Teboul J.L. (2014). Consensus on circulatory shock and hemodynamic monitoring. Task force of the European Society of Intensive Care Medicine. Intensive Care Med..

[2] Monnet X., Marik P.E., Teboul J.-L. (2016). Prediction of fluid responsiveness: An update. Ann. Intensive Care.

[3] Vincent J.-L., Weil M.H. (2006). Fluid challenge revisited. Crit. Care Med..

[4] Michard F., Boussat S., Chemla D., Anguel N., Mercat A., Lecarpentier Y., Richard C., Pinsky M.R., Teboul J.L. (2000). Relation between respiratory changes in arterial pulse pressure and fluid responsiveness in septic patients with acute circulatory failure. Am. J. Respir. Crit. Care Med..

[5] Vincent J.-L., Rhodes A., Perel A., Martin G.S., Della Rocca G., Vallet B., Pinsky M.R., Hofer C.K., Teboul J.-L., De Boode W.-P. (2011). Clinical review: Update on hemodynamic monitoring—A consensus of 16. Crit. Care.

[6] Bobbia X., Zieleskiewicz L., Pradeilles C., Hudson C., Muller L., Claret P.G., Leone M., de La Coussaye J.-E., Winfocus France Group (2017). The clinical impact and prevalence of emergency point-of-care ultrasound: A prospective multicenter study. Anaesth. Crit. Care Pain Med..

[7] Muller L., Toumi M., Bousquet P.-J., Riu-Poulenc B., Louart G., Candela D., Zoric L., Suehs C., de La Coussaye J.-E., Molinari N. (2011). An increase in aortic blood flow after an infusion of 100 ml colloid over 1 minute can predict fluid responsiveness: The mini-fluid challenge study. Anesthesiology.

[8] Blancas R., Martínez-González Ó., Ballesteros D., Núñez A., Luján J., Rodríguez-Serrano D., Hernández A., Martínez-Díaz C., Parra C.M., Matamala B.L. (2019). Lack of correlation between left ventricular outflow tract velocity time integral and stroke volume index in mechanically ventilated patients. Med. Intensiva.

[9] Maeder M.T., Karapanagiotidis S., Dewar E.M., Kaye D.M. (2015). Accuracy of Echocardiographic Cardiac Index Assessment in Subjects with Preserved Left Ventricular Ejection Fraction. Echocardiography.

[10] Bobbia X., Muller L., Claret P.-G., Vigouroux L., Perez-Martin A., de La Coussaye J.-E., Lefrant J., Louart G., Roger C., Markarian T. (2018). A New Echocardiographic Tool for Cardiac Output Evaluation: An Experimental Study. Shock.

[11] Grau-Mercier L., Coisy F., Markarian T., Muller L., Roger C., Lefrant J.-Y., Claret P.-G., Bobbia X. (2022). Can blood loss be assessed by echocardiography? An experimental study on a controlled hemorrhagic shock model in piglets. J. Trauma Acute Care Surg..

[12] Zieleskiewicz L., Claret P.-G., Muller L., de La Coussaye J.E., Lefrant J.Y., Schuster I., Roger C., Bobbia X. (2020). Global longitudinal strain changes during hemorrhagic shock: An experimental study. Turk. J. Emerg. Med..

[13] Mitchell C., Rahko P.S., Blauwet L.A., Canaday B., Finstuen J.A., Foster M.C., Horton K., Ogunyankin K.O., Palma R.A., Velazquez E.J. (2019). Guidelines for Performing a Comprehensive Transthoracic Echocardiographic Examination in Adults: Recommendations from the American Society of Echocardiography. J. Am. Soc. Echocardiogr..

[14] Temporelli P.L., Scapellato F., Eleuteri E., Imparato A., Giannuzzi P. (2010). Doppler echocardiography in advanced systolic heart failure: A noninvasive alternative to Swan-Ganz catheter. Circ. Heart Fail..

[15] Truijen J., Westerhof B.E., Kim Y.-S., Stok W.J., de Mol B.A., Preckel B., Hollmann M.W., van Lieshout J.J. (2018). The effect of haemodynamic and peripheral vascular variability on cardiac output monitoring: Thermodilution and non-invasive pulse contour cardiac output during cardiothoracic surgery. Anaesthesia.

[16] Yamada T., Tsutsui M., Sugo Y., Sato T., Akazawa T., Sato N., Yamashita K., Ishihara H., Takeda J. (2012). Multicenter study verifying a method of noninvasive continuous cardiac output measurement using pulse wave transit time: A comparison with intermittent bolus thermodilution cardiac output. Anesth. Analg..

[17] Kööbi T., Kaukinen S., Turjanmaa V.M. (1999). Cardiac output can be reliably measured noninvasively after coronary artery bypass grafting operation. Crit. Care Med..

[18] Distante A., Moscarelli E., Rovai D., L’Abbate A. (1980). Monitoring of changes in cardiac output by transcutaneous aortovelography, a non-invasive Doppler technique: Comparison with thermodilution. J. Nucl. Med. Allied. Sci..

[19] Mercado P., Maizel J., Beyls C., Titeca-Beauport D., Joris M., Kontar L., Riviere A., Bonef O., Soupison T., Tribouilloy C. (2017). Transthoracic echocardiography: An accurate and precise method for estimating cardiac output in the critically ill patient. Crit. Care.

[20] Feissel M., Michard F., Mangin I., Ruyer O., Faller J.P., Teboul J.L. (2001). Respiratory changes in aortic blood velocity as an indicator of fluid responsiveness in ventilated patients with septic shock. Chest.

[21] Shiran A., Adawi S., Ganaeem M., Asmer E. (2009). Accuracy and reproducibility of left ventricular outflow tract diameter measurement using transthoracic when compared with transesophageal echocardiography in systole and diastole. Eur. J. Echocardiogr..

